# CG and CHG Methylation Contribute to the Transcriptional Control of OsPRR37-Output Genes in Rice

**DOI:** 10.3389/fpls.2022.839457

**Published:** 2022-02-15

**Authors:** Chuan Liu, Na Li, Zeping Lu, Qianxi Sun, Xinhan Pang, Xudong Xiang, Changhao Deng, Zhengshuojian Xiong, Kunxian Shu, Fang Yang, Zhongli Hu

**Affiliations:** ^1^Chongqing Key Laboratory of Big Data for Bio Intelligence, Chongqing University of Posts and Telecommunications, Chongqing, China; ^2^State Key Laboratory of Hybrid Rice, College of Life Sciences, Wuhan University, Wuhan, China

**Keywords:** rice growth, DNA methylation, RNA-seq, circadian clock, output genes

## Abstract

Plant circadian clock coordinates endogenous transcriptional rhythms with diurnal changes of environmental cues. OsPRR37, a negative component in the rice circadian clock, reportedly regulates transcriptome rhythms, and agronomically important traits. However, the underlying regulatory mechanisms of OsPRR37-output genes remain largely unknown. In this study, whole genome bisulfite sequencing and high-throughput RNA sequencing were applied to verify the role of DNA methylation in the transcriptional control of OsPRR37-output genes. We found that the overexpression of *OsPRR37* suppressed rice growth and altered cytosine methylations in CG and CHG sequence contexts in but not the CHH context (H represents A, T, or C). In total, 35 overlapping genes were identified, and 25 of them showed negative correlation between the methylation level and gene expression. The promoter of the hexokinase gene *OsHXK1* was hypomethylated at both CG and CHG sites, and the expression of *OsHXK1* was significantly increased. Meanwhile, the leaf starch content was consistently lower in *OsPRR37* overexpression lines than in the recipient parent Guangluai 4. Further analysis with published data of time-course transcriptomes revealed that most overlapping genes showed peak expression phases from dusk to dawn. The genes involved in DNA methylation, methylation maintenance, and DNA demethylation were found to be actively expressed around dusk. A DNA glycosylase, namely ROS1A/DNG702, was probably the upstream candidate that demethylated the promoter of *OsHXK1*. Taken together, our results revealed that CG and CHG methylation contribute to the transcriptional regulation of OsPRR37-output genes, and hypomethylation of *OsHXK1* leads to decreased starch content and reduced plant growth in rice.

## Introduction

Plant DNA methylation occurs in the sequence context of CG, CHG, and CHH (where H is A, C, or T) (Zhang et al., 2006). DNA methylation is considered a stable epigenetic mark that can be transmitted across generations (Gehring, 2019). A specific DNA methylation state is also under the dynamic regulation by *de novo* methylation, maintenance of methylation, and active demethylation (Law and Jacobsen, 2010; Zhang et al., 2018). The function of DNA methylation in plant reproductive cells has been extensively studied, and this knowledge has deepened our understanding of the dynamic DNA methylation patterns during plant development (Zheng et al., 2019; Higo et al., 2020; Kim et al., 2021; Zhou et al., 2021a). In addition, DNA methylation reportedly has roles in regulating plant architecture, plant defense against rice black-streaked dwarf virus, and multiple agronomical traits (Zhang et al., 2015, 2020; Kawakatsu, 2020; Xu et al., 2020). However, whether DNA methylation plays a role in regulating circadian clock output genes remains unclear.

Circadian clock comprises multiple transcription-translation feedback loops, which function to improve plant environmental adaptation. Altered expression of circadian clock genes, such as *CIRCADIAN CLOCK ASSOCIATED1* (*CCA1*), can increase levels of plant growth and fitness (Dodd et al., 2005; Masuda et al., 2020). Meanwhile, expression amplitude of *CCA1* is associated with the CHH methylation level in the promoter region, which determines plant growth vigor (Ng et al., 2014). Recently, it was reported that the circadian clock genes *ZEITLUPE* and *TIMING OF CAB EXPRESSION 1* act downstream of DNA methyltransferases to control circadian rhythm (Tian et al., 2021). These results shed light on DNA methylation-mediated regulation of clock gene expression. *Pseudo-Response Regulators* (*PRR*s) are key components of transcription-translation feedback loops in plants and mediate the circadian regulation of clock output genes (Farre and Liu, 2013), including genes involved in the regulation of growth, flowering, abiotic stress, and yield-related traits (Li C. et al., 2020; Li N. et al., 2020; Sun et al., 2020; Wei et al., 2020; Liang et al., 2021). *OsPRR37* was primarily identified to delay the flowering time and increase grain yield and adaptation in rice (Koo et al., 2013; Liu et al., 2013, 2015; Yan et al., 2013; Gao et al., 2014; Fujino et al., 2019). A recent study further revealed a distinct role of *OsPRR37* in promoting flowering in the japonica variety Zhonghua 11 under natural long-day conditions (Hu et al., 2021). Although several output genes involved in the photoperiodic flowering pathway are used to explain the trait variations (Chen et al., 2021; Zhou et al., 2021c), the underlying mechanism of how *OsPRR37* regulates its output genes and multiple traits remains unclear.

Circadian regulation of plant transcriptome benefits the acute responses of plants to the daily fluctuating environment (Panter et al., 2019). Our previous study confirmed that OsPRR37 protein functions as a transcriptional repressor and confers expanded regulation of transcriptome rhythms (Liu C. et al., 2018). The regulation of circadian-regulated genes by DNA methylation in *Populus trichocarpa* suggested that DNA methylation contributes to the expression levels of clock output genes (Liang et al., 2019). Based on these results, we hypothesize that OsPRR37 uses DNA methylation as a pathway to regulate the transcription of its output genes. In the present study, we sought to determine whether and how DNA methylation regulates OsPRR37-output genes. To this end, whole-genome bisulfide sequencing (WGBS) and high-throughput RNA sequencing (RNA-seq) were applied to identify the overlapping genes, which were considered to be the OsPRR37-output genes regulated by DNA methylation. The available data of time-course transcriptomes were used to confirm the expression change of overlapping genes and to analyze the genes involved in DNA methylation pathways. Our results revealed that DNA methylation was an alternative medium for OsPRR37 to regulate the output genes and plant growth.

## Materials and Methods

### Plant Materials and Growth Conditions

The *OsPRR37* overexpression lines (OE5 and OE9) were described as previously reported (Liu C. et al., 2018). Briefly, *OsPRR37* overexpression lines were generated by overexpressing *OsPRR37* in an elite rice variety, namely Guangluai 4 (GL). NIL-*OsPRR37* is a nearly isogenic line in the GL background and contains the functional allele of *OsPRR37* from the elite variety Teqing. Rice growth phenotypes were obtained from the plants growing under natural long-day conditions in Wuhan University, Wuhan, China (30°54′01″N, 114°37′23″E). For WGBS and RNA sequencing, seeds of GL and OE5 were planted in a growth chamber (PRX-380B, Shanghai Guning Instrument Co., Ltd) for 15 days after germination. The growth chamber was set at 28°C under a 14-h light/10-h dark cycle with the light period of 6:00–20:00. The top most expanded leaves were harvested at 9:00, frozen in liquid nitrogen, and then stored at −80°C for DNA and RNA extraction. The same two biological replicates were applied to WGBS and RNA-seq.

### Bisulfite-Seq Library Generation and Sequencing

Briefly, total genomic DNA of rice leaves was extracted using the cetyltrimethylammonium bromide method (Doyle, 1987). The DNA concentration and quality were estimated using NanoDrop 2000 spectrophotometer (NanoDrop Technologies, Wilmington, DE, United States), Qubit 3.0 fluorometer (Life Technologies, Carlsbad, CA, United States), and 1.0% agarose gel electrophoresis. Then, 2-μg genomic DNA spiked with 5-ng unmethylated Lambda DNA (Promega, Madison, WI, United States) was fragmented by sonication to generate fragments measuring 300–500 bp. These fragments were then ligated with 5-methylcytosine-modified adapters and subjected to bisulfide conversion using the ZYMO EZ DNA Methylation-Gold Kit (Zymo Research, Irvine, CA, United States). The bisulfide-converted DNA was purified, recycled, and then amplified by PCR with 10 cycles using KAPA HiFi HotStart Uracil + ReadyMix (Kapa Biosystems, Wilmington, MA, United States) and Illumina 8-bp index primers. The WGBS libraries were analyzed using the Bioanalyzer 2100 system (Agilent Technologies, CA, United States) and sequenced on Illumina NovaSeq 6000 with a paired-end sequencing length of 150 bp (PE150) at Frasergen Bioinformatics Co., Ltd (Wuhan, China). The percentage of cytosines sequenced at cytosine reference positions in the lambda genome was considered to reflect the overall sodium bisulfite non-conversion rate.

### Bisulfite-Seq Data Processing and Analysis

Quality control of WGBS data was performed using FastQC (version 0.11.9, Babraham Bioinformatics, United Kingdom). Sequencing adapters and low-quality reads were removed using Trimmomatic (Bolger et al., 2014). The trimmed reads were then aligned and mapped to the rice reference genome of Nipponbare (MSU_v7.0) using Bismark (Krueger and Andrews, 2011). The percentage methylation level was calculated by mC/(mC + umC), where mC and umC represent the number of methylated and unmethylated reads, respectively. Only the CG/CHG/CHH sites with a read coverage of ≥ 5 across all samples were used for differential methylation analyses. Differentially methylated regions (DMRs) were identified using the R package “dmrseq” (Korthauer et al., 2019). Regions with a *q*-value < 0.05, number of CG/CHG/CHH sites ≥ 5 and methylation difference > 20% were defined as DMRs. DMR distribution on rice chromosomes was plotted using Circos (version 0.69) (Krzywinski et al., 2009). DMRs were annotated using ChIPseeker package (Yu et al., 2015). Methylation status along the genomic regions of DMRs ± 20 kb was plotted using pyGenomeTracks (version 3.6) (Lopez-Delisle et al., 2021).

### RNA Library Generation and Sequencing

Total RNA from rice leaves was extracted using TRIzol Reagent (Invitrogen, CA, United States) for RNA sequencing. RNA purity and integrity were analyzed using a NanoDrop 2000 spectrophotometer (NanoDrop Technologies, Wilmington, DE, United States) and the Bioanalyzer 2100 system (Agilent Technologies, CA, United States). RNA contamination was assessed by 1.5% agarose gel electrophoresis. A total of 1 μg of RNA per sample was used as the input material for library preparation. The mRNA was purified from the total RNA using poly-T oligo-attached magnetic beads. Sequencing libraries were generated from the purified mRNA using the VAHTS Universal V6 RNA-seq Library Kit for MGI (Vazyme, Nanjing, China) following the manufacturer’s recommendations with unique index codes. The library quantification and size were assessed using a Qubit 3.0 fluorometer (Life Technologies, Carlsbad, CA, United States) and Bioanalyzer 2100 system (Agilent Technologies, CA, United States). Subsequently, sequencing with a paired-end sequencing length of 150 bp (PE150) was performed on the MGI-SEQ 2000 platform (MGI Tech Co., Ltd. Shenzhen, China) by Frasergen Bioinformatics Co., Ltd (Wuhan, China).

### Bioinformatics Analysis of RNA Sequencing and Microarray Data

Sequencing adapters and low-quality reads were removed with fastp (version 0.20.1) (Chen et al., 2018), and the quality of raw reads was evaluated with FastQC (version 0.11.9, Babraham Bioinformatics, United Kingdom). The remaining clean reads were mapped to the rice reference genome of Nipponbare (MSU_v7.0) using Hisat2 (version 2.1.0) (Kim et al., 2019). Mapping statistics were generated using Samtools (version 1.11) (Li H. et al., 2009). TPMCalculator was used to count the reads mapped to individual genes as well as to measure gene expression levels by calculating transcripts per million (TPM) read values (Vera Alvarez et al., 2019). Differentially expressed genes (DEGs) were identified using DESeq2 (Love et al., 2014). Gene Ontology (GO) enrichment analysis was performed using the GO annotation file MSU7.0 gene ID (TIGR) of agriGO v2.0 and clusterProfiler 4.0 (Tian et al., 2017; Wu et al., 2021). KEGG enrichment analysis was performed using KOBAS 3.0, and the output data were plotted using clusterProfiler 4.0 (Bu et al., 2021). Gene symbols with known or unknown function were annotated using MBKbase-rice database^1^, funRiceGenes database^2^, and China Rice Data Center^3^ (Yao et al., 2018; Peng et al., 2020). The raw RNA-seq data of time-course transcriptomes, which were downloaded from NCBI-GEO database (GSE114188), were reanalyzed as per the RNA-seq data processing pipeline in this study. The corresponding time-course samples of GL and OE5 comprise six time points (4:00, 8:00, 12:00, 16:00, 20:00, and 0:00) with three replicates at 45 days growth under natural long-day conditions. Statistical significance of different expressions was evaluated by unpaired Student’s *t*-test at each time point. The microarray data were obtained by GSE19024 on NCBI-GEO (Wang et al., 2010). The corresponding tissue samples of interest were described in Supplementary Table 1, which were the subset of samples in a previous study (Wang et al., 2010). Before being used to plot the heatmap, the signal values of biological and technical replicates for the same tissue were averaged.

### Quantitative RT-PCR and Starch Content Determination

Quantitative RT-PCR was conducted with the same protocol as previously reported (Liu et al., 2015). The PCR primer sequences were 5′-TGACAAAGCCTAGTACAAATAAGGAGAG-3′ and 5′-CAGTGCTGTGCAGGATGAAATG-3′. Approximately, 0.2 g of fresh leaf samples were weighed before estimating the starch content. Starch content was determined according to previously published protocols (Smith and Zeeman, 2006).

## Results

### Rice Growth Was Repressed by Overexpressing *OsPRR37*

**FIGURE 1:**
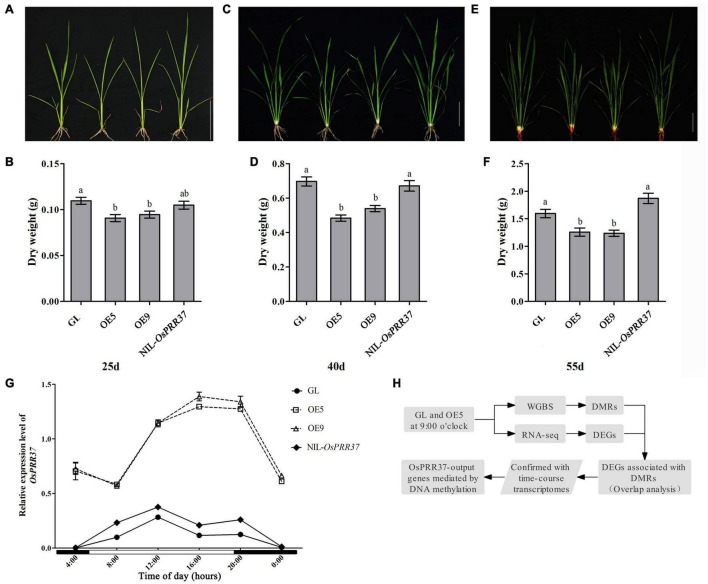
Characterization of rice growth and *OsPRR37* expression rhythms. Rice growth morphology and dry weight were documented at 25 days **(A,B)**, 40 days **(C,D)**, and 55 days **(E,F)** after sowing the seeds. The different lower-case characters above bars represent the significant difference level of *P* < 0.05. **(G)** The expression levels of *OsPRR37* detected by quantitative RT-PCR. The exact time of a natural long-day condition was indicated under the *X* axis. **(H)** Simplified workflow of the present study. OE5 and OE9 are two independent transgenic lines.

During the field trails, we observed that rice growth was retarded in *OsPRR37* overexpression lines (OE). To investigate the effects of *OsPRR37* overexpression on rice growth, we record the morphology and dry weight of GL, OE5, OE9, and NIL-*OsPRR37* at 25, 40, and 55 days after sowing the seeds. The growth of OE5 and OE9 was significantly repressed compared to the growth of GL and NIL-*OsPRR37* on these days (Figures 1A–F). These results suggest that natural loss-of-function and gain-of-function alleles of *OsPRR37* showed a comparable growth rate during the vegetative growth period. Then, the diurnal expression profile of *OsPRR37* was monitored over a day using quantitative RT-PCR. *OsPRR37* was identified as having similar expression rhythms in GL and NIL-*OsPRR37* as their peak expression phase was around 12:00. Conversely, the expression of *OsPRR37* in OE5 and OE9 was much higher and showed altered rhythms with the peak expression phase around 4:00 (Figure 1G). These results suggested that the overexpression of *OsPRR37* changed its diurnal rhythms and repressed rice growth. In a previous study, it was reported that *OsPRR37* widely regulates output genes and particularly suppresses output genes with phases around 9:00 (Liu C. et al., 2018). To investigate whether DNA methylation associated with OsPRR37 regulates the output genes, samples of GL and OE5 at 9:00 were subjected to WGBS and RNA-seq. The workflow of this study is shown in Figure 1H.

### Whole-Genome Bisulfide Sequencing and RNA Sequencing Quality Assessment and Alignment

Whole-genome bisulfide sequencing and RNA-seq were used to investigate the role of DNA methylation in the transcriptional regulation of OsPRR37-output genes. WGBS generated 48,752,185 and 63,773,173 raw reads for the two GL replicates and 59,530,161 and 56,527,166 raw reads for the two OE5 replicates. After quality control filtration, 45,246,229 and 59,675,582 clean reads remained for GL, and 56,065,476 and 53,133,046 clean reads remained for OE5. The clean reads ratio ranged from 92.8 to 94.2%. The average percentage of Q30 and GC content for the four sequencing libraries was 92.7 and 22.9%, respectively (Supplementary Table 2). Of those clean data, 53.4% (GL-1), 51.7% (GL-2), 54.2% (OE5-1), and 52.2% (OE5-2) were uniquely mapped to the rice genome (Supplementary Table 3). Overall, 30,872,222 CG sites, 27,422,379 CHG sites, and 104,533,760 CHH sites were identified with sequencing coverage range from 53.1 to 69.4% (Supplementary Table 4). Among these, 10.6%–13.0% CG sites, 7.7–9.7% CHG sites, and 4.0–4.9% CHH sites were methylated (Supplementary Table 5). The same samples of WGBS were used in RNA-seq to obtain comparable data. In total, RNA-seq generated 23,559,582 (GL-1), 25,090,638 (GL-2), 25,898,111 (OE5-1), and 26,350,196 (OE5-2) clean read pairs (Supplementary Table 6). The percentages of Q30 ranged between 84.9 and 86.1%. Of these clean read pairs, 90.0 to 90.7% were mapped to the reference genome of rice (Supplementary Table 7). These data were sufficient and reliable for subsequent differential methylation and expression analysis.

### Overexpressing *OsPRR37* Altered Global CG and CHG Methylation

**FIGURE 2:**
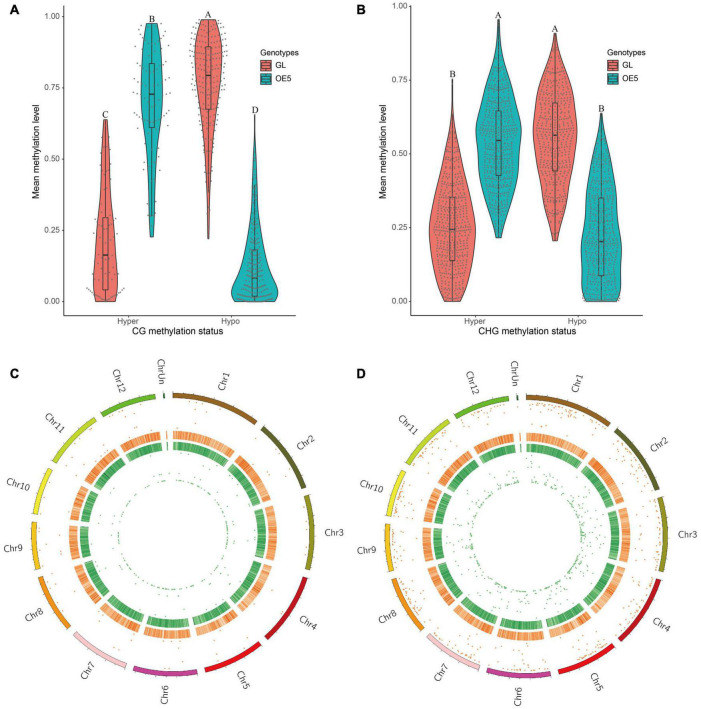
Identification and analysis of differentially methylated regions. Comparisons of hypermethylation and hypomethylation levels in GL and OE5 were plotted for both CG **(A)** and CHG **(B)** sequence contexts. Different letters above violin plots represent significant differences at *P* < 0.01 as revealed by one-way ANOVA analysis (Tukey’s multiple comparison test). DMR distribution on rice chromosomes in CG **(C)** and CHG **(D)** sequence contexts is shown. From outer to inner layers, the circular plots represent chromosomes, hyper-DMR distribution (the more outward means higher significance), heatmap of GC content (deeper red colors indicate higher GC content), heatmap of gene density (deeper green colors indicate higher gene density), and hypo-DMR distribution (greater proximity to the center of the circle indicates higher significance).

To identify DMRs, three cytosine sequence contexts (CG, CHG, and CHH) were separately applied to differential methylation analysis. Genomic regions with a *q*-value of < 0.05 and a differential methylation level of > 20% were considered as DMRs. A total of 321 and 949 DMRs were found in CG (DMR-CG) and CHG (DMR-CHG) sequence contexts, respectively. However, no DMRs were identified in the CHH sequence context. Among DMR-CG, 90 were hypermethylated and 231 were hypomethylated (Figure 2A). Conversely, among DMR-CHG, 480 were hypermethylated and 469 were hypomethylated (Figure 2B). These data revealed a higher proportion of hypomethylated DMR-CG (72.0%) than DMR-CHG (49.4%). Furthermore, the significance of methylated DMRs across the 12 chromosomes found that DMRs were evenly distributed on the rice genome and were of high significance (Figures 2C,D).

**FIGURE 3:**
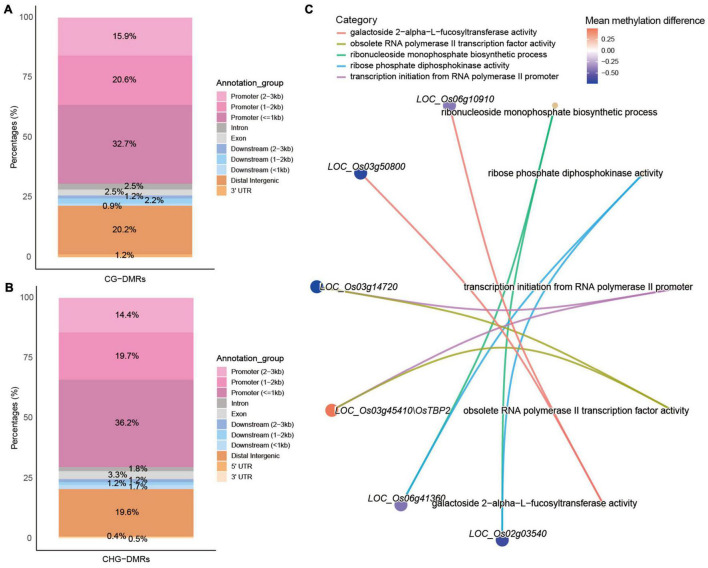
Characterization of DMR-associated genes. The distributions of CG-DMRs **(A)** and CHG-DMRs **(B)** summarized according to the annotated genomic location. The order of the stacking bar charts is consistent with that of the legends on the right. **(C)** The network between genes and enriched GO terms. Genes are indicated as MSU loci or gene symbols. GO categories are displayed in the name of GO terms.

To obtain DMR-associated genes (DMGs), DMR-CG and DMR-CHG were both annotated with the R package “ChIPseeker.” The result showed that 32.7%, 20.6%, and 15.9% of DMR-CG were located in the promoter region at ≤1 kb, 1–2 kb, and 2–3 kb upstream of transcription start site, respectively (Figure 3A). The distal intergenic region accounted for 20.2% of DMR-CG. Conversely, only a small fraction of DMR-CG was annotated within Intron (2.5%), Exon (2.5%), Downstream (≤1 kb: 0.9%, 1–2 kb: 2.2%, and 2–3 kb: 1.2%) and 3′UTR (1.2%). This means that most DMR-CG (69.2%) were located in the promoter region (Figure 3A). Similarly, 70.3% of DMR-CHG were located in promoter region (Figure 3B). These results supported the notion that cytosine methylation majorly occurred in the promoter sequence. Then, we performed functional enrichment analysis with these DMGs. The network of five most enriched GO ontologies for DMR-CG-associated genes showed that LOC_Os02g03540 and LOC_Os06g41360 enable ribose phosphate diphosphokinase activity and are involved in ribonucleoside monophosphate biosynthetic process (Figure 3C). LOC_Os03g45410/OsTBP2 and LOC_Os03g14720 enable obsolete RNA polymerase II transcription factor activity and are involved in transcription initiation from the RNA polymerase II promoter. LOC_Os03g45410/OsTBP2 was reported to be a TATA-binding protein, which interacts with the transcription factor IIB (Zhu et al., 2002). Interestingly, LOC_Os03g14720 is a putative transcription initiation factor IIF. These results highlighted that DMR-CG-associated genes mainly function in gene transcription regulation. However, no GO term was found to be significantly enriched for DMR-CHG-associated genes.

### Differentially Expressed Gene Analysis

**FIGURE 4:**
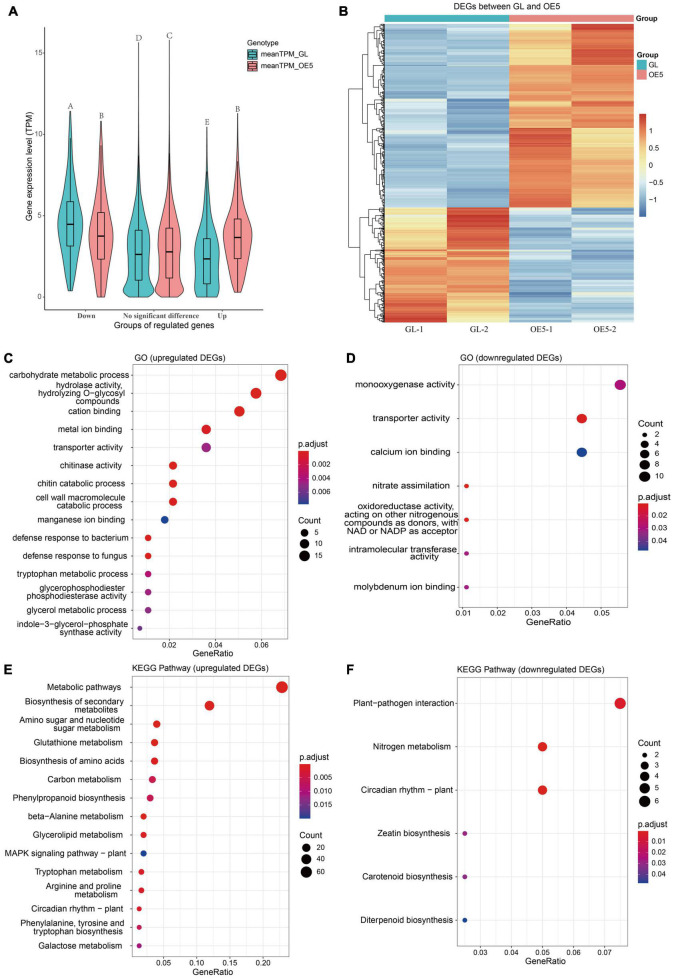
Expression and functional enrichment of differentially expressed genes. **(A)** A comparison of mean expression levels of upregulated, no significant difference, and downregulated genes between GL and OE. Different letters above violin plots represent significant differences at *P* < 0.01 as revealed by one-way ANOVA analysis (Tukey’s multiple comparison test). **(B)** The heatmap of 457 upregulated and 286 downregulated DEGs. The TPM value of DEG was scaled by row with the “pheatmap” package in R. Dotplots of significant GO terms for upregulated **(C)** and downregulated **(D)** DEGs. Dotplots of significant KEGG pathways for upregulated **(E)** and downregulated **(F)** DEGs. GO terms and KEGG pathways with adjusted *P*-value < 0.05 were considered as significant, and if the number of significant terms or pathways was > 15, only 15 terms or pathways were plotted.

Although *OsPRR37* overexpression altered the methylation of >1,000 DMGs, the number of transcriptionally regulated DMGs remains unknown. Samples of GL and OE5 were subjected to RNA-seq to profile the genome-wide gene expressions. The overall gene expression level was slightly higher for OE5 than for GL (Supplementary Figure 1), whereas the expression correlation between samples was ranged from 0.9804 to 0.9929 (Supplementary Figure 2). Genes with low expression (sum of TPM being < 2 in both GL and OE5) were filtered out. A total of 743 DEGs (| log_2_FC| > | log_2_1.5|, adjusted *P*-value < 0.05) were identified between GL and OE5 (Supplementary Figure 3). Among these DEGs, 286 (38.5%) were downregulated and 457 (61.5%) were upregulated (Figures 4A,B). The increment of the mean TPM for upregulated DEGs was larger than the decrement of the mean TPM for downregulated DEGs (Figure 4A). The expression of DEGs in the two replicates was similar so that DEGs are robust to be further analyzed (Figure 4B).

GO enrichment analysis found that DEGs are instrumental in metal ion binding, transporter activity, and chitinase activity and are mainly involved in carbohydrate metabolic process, chitin catabolic process, defense response to bacterium and fungus, and nitrate assimilation, among other functions (Figures 4C,D). The KEGG pathway enrichment analysis showed that upregulated DEGs participate in amino sugar and nucleotide sugar metabolism, carbon metabolism, glycerolipid metabolism, MAPK signaling pathway, and circadian rhythm, among other roles. Downregulated DEGs are mainly involved in plant–pathogen interaction, nitrogen metabolism, and circadian rhythm (Figures 4E,F).

### Characterization of Overlapping Genes Between DMR-Associated Genes and Differentially Expressed Genes

**FIGURE 5:**
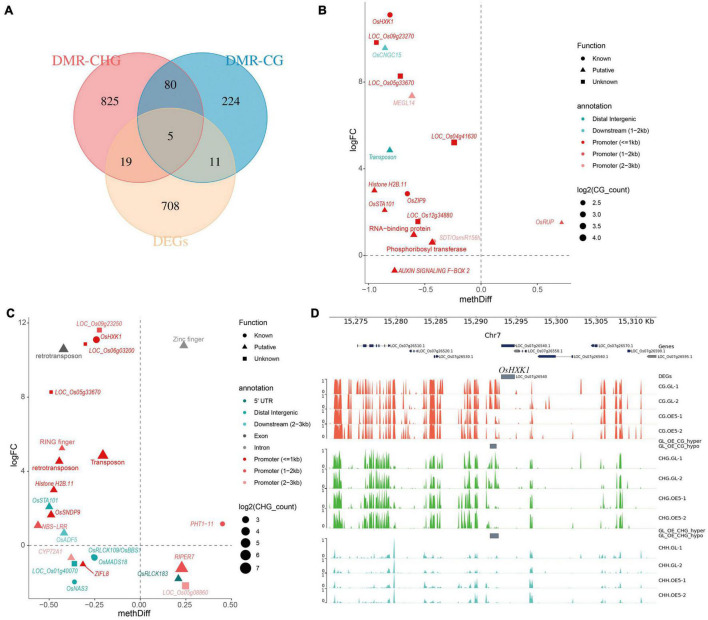
Overlap analysis between DMGs and DEGs. **(A)** The distribution of overlapping genes between DMGs and DEGs. Scatter plots of the correlation between methylation difference and expression fold change for overlapping genes in CG **(B)** and CHG **(C)** contexts. **(D)** Visualization of methylation status for the representative gene *OsHXK1*.

Overlap analysis between DMGs and DEGs was performed to identify transcriptionally regulated DMGs. In total, 35 genes were found to be shared between DMG and DEG sets. Among these, five DEGs were found to be differentially methylated in both CG and CHG sequence contexts, whereas 19 DEGs were uniquely shared with DMR-CHG and 11 DEGs were uniquely shared with DMR-CG (Figure 5A). The correlation between methylation difference and expression fold-change of overlapping genes was investigated. Consequently, 14 of 16 genes (87.5%) showed negative correlation between expression and CG methylation, and 14 of 16 genes (87.5%) were methylated in promoter regions (Figure 5B). In contrast, 16 out of 24 genes (66.7%) showed a negative correlation between expression and CHG methylation, and 15 of 24 genes (62.5%) were methylated in promoter regions (Figure 5C). After removing the redundant genes, in total, 25 genes showed negative correlation between expression and cytosine methylation level (Figures 5B,C). Functional annotation with the MBKbase, funRiceGenes database and China Rice Data Center identified seven genes with known function: *OsHXK1* (*LOC_Os07g26540*), *OsZIP9* (*LOC_Os05g39540*), *SDT/OsmiR156h* (*LOC_Os06g44034*), *OsMADS18* (*LOC_Os07g41370*), *OsPT11* (*LOC_Os01g46860*), *OsRLCK109/OsBBS1* (*LOC_Os03g24930*), and OsNAS3 (*LOC_Os07g48980*). Among these genes, *OsHXK1* showed the highest negative correlation between methylation difference and expression fold-change (Figures 5B,C). Detailed methylation status indicated that the CG and CHG sites in the promoter region of *OsHXK1* were both hypomethylated (Figure 5D).

### Diurnal Rhythms and Functional Characterization of Overlapping Genes

**FIGURE 6:**
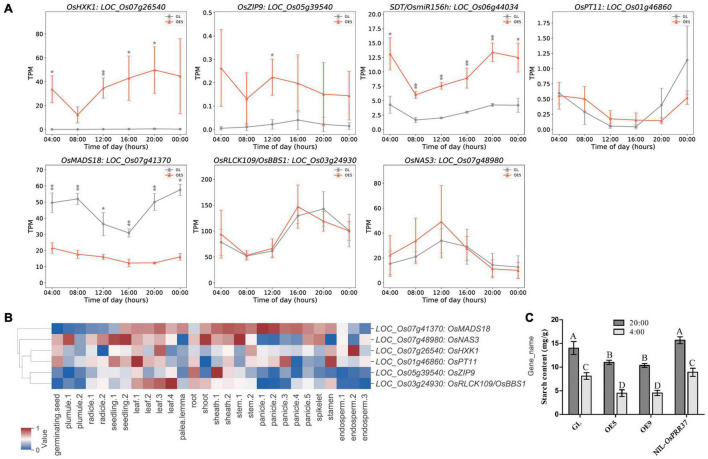
Diurnal and tissue-specific expression analysis of the known overlapping genes. **(A)** Diurnal expression of the known overlapping genes between DMGs and DEGs. The asterisks above curves mean significance difference at *P* < 0.05 (one asterisk) and at *P* < 0.01 (two asterisks). **(B)** Tissue-specific expression analysis of known overlapping genes. **(C)** The measure of the starch content in rice leaves at the end (20:00) and beginning (4:00) of the day. Different letters above bars represent significant differences at *P* < 0.01 as revealed by one-way ANOVA analysis (Bonferroni’s multiple comparison test). Values represent the means ± SD of three replicates.

As OsPRR37 is a key component in the rice circadian clock, diurnal rhythms of overlapping genes were further investigated with the reported time-course RNA-seq data of leaf samples at 45 days growth (Liu C. et al., 2018). Among the 35 overlapping genes, 29 were observed to be differentially expressed with at least one timepoint, which confirmed the identification of DEGs in this study (Figure 6A and Supplementary Figure 4). We also found that 31 overlapping genes showed diurnal rhythms, and 27 were observed to show a peak expression phase of 16:00–04:00. These data indicated that most differentially methylated DEGs were under circadian control and tended to function from dusk to dawn. Further investigation of the seven reported overlapping genes revealed that four of them showed significant differences in their expression across different time points of the day (Figure 6A). Interestingly, *OsHXK1*, *SDT*/*OsmiR156h*, *OsMADS18*, and *OsPT11* were diurnally expressed and showed a peak expression phase of 20:00–4:00, indicating that they predominantly function during the night. *SDT*/*OsmiR156h* can modulate the rice yield, plant architecture, and seed dormancy by targeting *Ideal Plant Architecture1* (*IPA1*) (Jiao et al., 2010; Miao et al., 2019). Its continuously high expression suggested the involvement of the *SDT*/*OsmiR156h*-*IPA1* module in *OsPRR37*-mediated rice growth regulation. Except for *SDT*/*OsmiR156h*, the other six genes were mapped in the microarray data of Zhenshan97 tissues (Wang et al., 2010). *OsMADS18* was widely expressed in the tissues of seedling, leaf, shoot, sheath, stem, and panicle, which is in line with its function in flowering signal transduction (Figure 6B; Fornara et al., 2004; Yin et al., 2019). The significant repression of *OsMADS18* can partly explain the delayed growth and flowering (Figure 6A). *OsNAS3* encodes a nicotianamine synthase that is important for Fe homeostasis (Aung et al., 2019). Our result showed that *OsNAS3* was widely expressed in germinating seed, plumule, radicle, seedling, leaf, root, shoot, sheath, stem, panicle, and spikelet. *OsRLCK109*/*OsBBS1* was diurnally expressed with a peak phase of 16:00–20:00 and was highly expressed in leaf, root and sheath to regulate leaf senescence and salt stress responses (Zeng et al., 2018). *OsZIP9* was mainly expressed in the root and sheath to uptake zinc for rice growth (Tan et al., 2020; Yang et al., 2020). However, even though *OsPT11* is a rice phosphate transporter that regulates phosphate uptake and transport (Paszkowski et al., 2002; Yang et al., 2012), it was highly expressed in many tissues, such as geminating seed, radicle, root, leaf, sheath, stamen, endosperm, and panicle. The role of *OsZIP9* and *OsPT11* in coordinating ion uptake and rice growth needs to be further confirmed.

*OsHXK1* was identified to be highly expressed in the germinating seed, leaf, stem, stamen, and endosperm (Figure 6B). This expression pattern is in close agreement with a previous study wherein *OsHXK1* was reported to regulate reactive oxygen species in rice anthers (Zheng et al., 2019) and knockout of *OsHXK1* improved rice photosynthetic efficiency and yield (Zheng et al., 2021). In plant leaves, starch is accumulated during the day and consumed by respiration at night, and therefore, the starch content can indicate the strength of photosynthesis. To investigate whether the photosynthesis product was altered by the significantly elevated expression of *OsHXK1*, we compared the total starch content in GL, OE5, OE9, and NIL-*OsPRR37* leaves at the ending (20:00) and beginning (4:00) of the day. We found the total starch content to be significantly lower in OE5 and OE9 than in GL and NIL-*OsPRR37* at both time points (Figure 6C). The low starch content in OE lines resulted in energy deficit, consequently causing repressed rice growth (Figure 1). These results revealed that the enhanced expression of *OsHXK1* by hypomethylation decreased starch content and rice growth, thus suggesting that *OsHXK1* is a key output gene applied by *OsPRR37* to regulate rice growth.

### Diurnal Expression Analysis of DNA Methylation Related Genes

**FIGURE 7:**
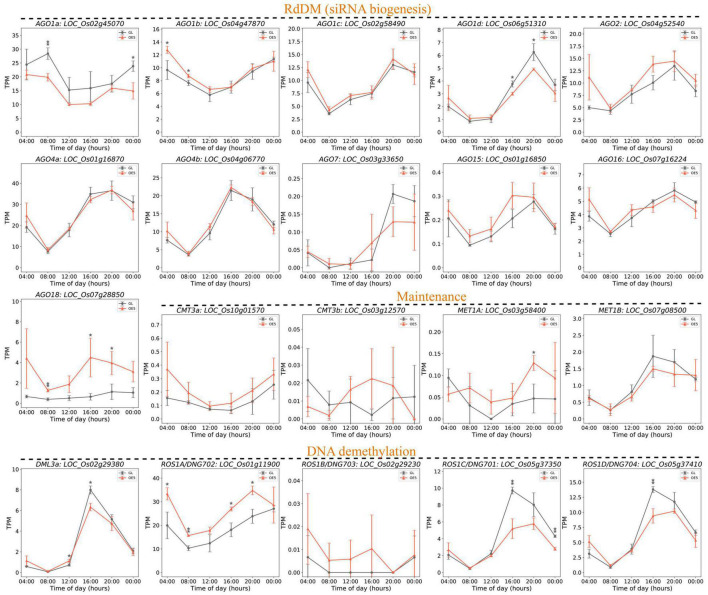
Diurnal expression patterns of DNA methylation-related genes. Eleven *AGO* family members in the RdDM pathway, four genes for methylation maintenance, and five for DNA demethylation were presented with data of time-course transcriptomes. The three groups of genes are arranged under horizontal dash lines with group names of RdDM (siRNA biogenesis), Maintenance, and DNA demethylation. The other five DNA methylation genes and 11 siRNA biogenesis genes in the RdDM pathway are shown in Supplementary Figure 5. The asterisks above curves mean significance difference at *P* < 0.05 (one asterisk) and *P* < 0.01 (two asterisks).

DNA methylation patterns are decided by coordinated regulation of DNA methylation and demethylation pathways. To explore the upstream genes of DMGs, the genes involved in DNA methylation were profiled with time-course RNA-seq data. In total, based on a recent study, 36 genes were grouped into the RNA-directed DNA methylation (RdDM) pathway, methylation maintenance, and DNA demethylation (Sun et al., 2021). Interestingly, most of them (26 genes) exhibited a peak expression phase of 16:00–20:00 (Figure 7 and Supplementary Figure 5), indicating that DNA methylation, methylation maintenance, and DNA demethylation are particularly active around dusk. Among these, several genes encoding Argonaute (AGO) proteins are altered, including the downregulated *AGO1a* and *AGO1d* and the upregulated *AGO1b* and *AGO18* (Figure 7). The miR168-*AGO1* module can regulate multiple miRNAs to improve yield, reduce flowering time, and enhance immunity (Wang et al., 2021). Meanwhile, AGO18 sequesters miR168 to alleviate the repression of rice AGO1 (Wu et al., 2015), and a regulation module of *miR168a*–*OsAGO1*/*OsAGO18–*-*miRNAs*-target genes was proposed to regulate agronomically important traits (Zhou et al., 2021a). These results combined with our data suggest a causal link between rice growth repression and the altered module of *miR168a*-*OsAGO1*/*OsAGO18*.

A relatively similar expression of methylation maintenance genes was observed between GL and OE5. Conversely, four out of five genes of DNA demethylation showed significantly different expression in GL and OE5 (Figure 7). Interestingly, the expression of *ROS1C*/*DNG701* and *ROS1D*/*DNG704* was suppressed, whereas that of *ROS1A*/*DNG702* was increased. These three genes were recently reported to demethylate DNA in the gamete and zygote, which is crucial for zygote gene expression and development (Zhou et al., 2021b). In addition, a mutation in *ROS1A*/*DNG702* can generally lead to the increase of CG and CHG but not of CHH hypermethylation on genomes of rice endosperms (Liu J. et al., 2018). This result strongly corroborates our data wherein DMRs were only identified in CG and CHG sequence contexts, and because the expression of *ROS1A*/*DNG702* was increased in OE5, markedly more hypo-DMRs (55.1%) and upregulated DEGs (61.5%) were observed (Figure 7). The effect of *ROS1A*/*DNG702* may be counteracted by the decreased expression of *ROS1C*/*DNG701* and *ROS1D*/*DNG704*. Taken together, we believe that ROS1A/DNG702 was the upstream protein that demethylated the promoter regions of *OsHXK1* and enhanced its expression, thus leading to decreased starch content and reduced rice growth.

## Discussion

### Circadian Rhythm of *OsPRR37* Is Important for Rice Growth

The endogenous expression period of clock genes is crucial for a plant to match the light–dark cycle. If correctly matched, the plant circadian system will enhance photosynthetic carbon fixation and growth (Dodd et al., 2005). Our results found that the circadian expression pattern of *OsPRR37* in OE5 and OE9 was significantly different from that in GL and NIL-*OsPRR37* (Figure 1). Although GL contains a loss-of-function allele of *OsPRR37*, the circadian rhythm and plant growth observed were similar between GL and NIL-*OsPRR37* (Figure 1). Moreover, GL and NIL-*OsPRR37* showed no significant difference in the starch content (Figure 6C). These results confirmed that disturbing circadian rhythm of *OsPRR37* decreased starch content and plant growth.

### Input and Output Pathways for *OsPRR37*

The regulatory network of transcription-translation feedback loops in the core circadian clock is well drawn based on exciting results of research on clock genes (Nakamichi, 2020). However, the inputs and outputs of the circadian clock remain unclear. The photosynthetic endogenous sugar levels provide metabolic entrainment to the circadian clock system through the morning-phased gene *PRR7*, the homolog of *OsPRR37* in Arabidopsis (Haydon et al., 2013). A recent study reported that PRR7 mediates the circadian input to the promoter of CCA1 in the shoots (Nimmo and Laird, 2021). These results indicate an entrainment route of sugar–PRR7–CCA1. In rice, sugars suppress *OsCCA1* expression while OsCCA1 regulates *IPA1* expression to mediate panicle and grain development (Wang et al., 2020). Our results suggested that the *SDT*/*OsmiR156h*-IPA1 module was involved in modulating OsPRR37-mediated rice growth. Based on these results, whether and how sugar–*OsPRR37*–*OsCCA1*–*SDT*/*OsmiR156h*–*IPA1* comprises an integrated pathway need more evidence in the future. Furthermore, the enrichment analysis found that upregulated genes were enriched in carbohydrate metabolic process (Figure 4C), amino sugar and nucleotide sugar metabolism, and carbon metabolism pathways (Figure 4E). These results suggested that sugar and carbon metabolism pathways are altered by *OsPRR37* overexpression. Meanwhile, several downregulated genes are enriched in nitrate assimilation (Figure 4D) and nitrogen metabolism (Figure 4F), suggesting that nitrate assimilation and metabolism would be other pathways coordinated by OsPRR37 to affect plant growth.

### The Role of Differentially Methylated OsPRR37-Output Genes

Epigenetic modifications are closely associated with alterations in chromatin structure, such as histone modification and DNA methylation. Rhythmic transcription of Arabidopsis clock genes was considered to be regulated by rhythmic histone modification (Song and Noh, 2012). However, to our knowledge, there has been no research on the role of DNA methylation in regulating clock output genes. OsPRR37 was believed to repress morning-phased output genes and indirectly activate evening-phase output genes (Liu C. et al., 2018). In the present study, as we focused on samples in the morning (9:00), and our primary goal was to identify the key overlapping genes that were downregulated by OsPRR37. In this process, we hoped to get some insight into how OsPRR37 is associated with DNA methylation pathways so as to directly repress the morning-phased output genes. However, the results showed that 25 out of 35 overlapping genes were upregulated, and the expression levels of 22 genes were negatively correlated with methylation levels (Figures 5B,C). These results supported our hypothesis that DNA methylation contributed to the regulation of OsPRR37-output genes, but the dynamic methylation of these output genes is probably under an indirect regulation of OsPRR37. In other words, the differentially methylated output genes are in the most downstream of OsPRR37, such as *OsHXK1*, *SDT*/*OsmiR156h*, and *OsMADS18*, which are more directly to regulate rice growth, flowering, and yield.

### The Hierarchical Regulation Network of *OsPRR37*

Different members of PRRs are supposed to function at their specific times of the day to repress clock output genes (Farre and Liu, 2013). Accumulating evidence has indicated that PRRs interact with other proteins to regulate the transcription of output genes. The B-box (BBX)-containing proteins BBX19 and BBX18 can physically interact with PRR9, PRR7, and PRR5 in a precise temporal order from dawn to dusk, thus cooperatively regulating the output genes (Yuan et al., 2021). OsPRR73 interacts with histone deacetylase 10 (HDAC10) to co-repress *OsHKT2;1*, a plasma membrane-localized Na(+) transporter, and confers salt stress tolerance to rice (Wei et al., 2020). The OsPRR37 protein can interact with Ghd8 and NF-YCs, which form an alternative OsNF-Y heterotrimer to affect Hd1-mediated regulation of *Hd3a* and flowering (Goretti et al., 2017). The distinct role of *OsPRR37* in the ZH11 background indicated that OsPRR37 can associate with different partners to perform different functions (Hu et al., 2021). Moreover, the 35 identified differentially methylated DEGs accounted for only a small proportion of DEGs (Figure 5A). These results draw a map of the hierarchical regulation network for OsPRR37 and thus put forward an interesting question about the partners of OsPRR37 with which it regulates the large amount of remaining DEGs. Nevertheless, differentially methylated DEGs are the key candidates to regulate rice growth.

Epigenetic marks that modulate the expression of genes behind the traits of interest have potential applications in crop enhancement (Kakoulidou et al., 2021). With the development of multi-omics technologies and related data processing pipelines (Feng et al., 2021; Iqbal et al., 2021), the hierarchical regulation network of the circadian clock will be gradually parsed and applied to improve rice traits. Recently, the representative role of OsPRR37 in the control of photoperiodic flowering was systematically reviewed (Chen et al., 2021; Zhou et al., 2021c). However, the underlying mechanism of how OsPRR37 regulates its output genes to affect multiple agronomic traits remains unclear. By integrative analysis of WGBS and RNA-seq data, our results revealed that DNA methylation contributes to the regulation of OsPRR37-output genes, which provides an alternative strategy to improve plant growth through epigenetic modulation of OsPRR37-output genes.

## Data Availability Statement

The datasets presented in this study can be found in online repositories. The name of the repository and accession number can be found below: GEO, NCBI: GSE192416.

## Author Contributions

CL designed the study, carried out most of the data analysis, and wrote the manuscript. NL performed the qRT-PCR and determined the starch content. ZL, QS, XP, XX, CD, and ZX performed the data analysis of time-course transcriptomes and tissue-specific microarray data. KS, FY, and ZH provided insightful suggestions on data analysis and writing of manuscript. All authors have read and agreed to the submitted version of the manuscript.

## Conflict of Interest

The authors declare that the research was conducted in the absence of any commercial or financial relationships that could be construed as a potential conflict of interest.

## Publisher’s Note

All claims expressed in this article are solely those of the authors and do not necessarily represent those of their affiliated organizations, or those of the publisher, the editors and the reviewers. Any product that may be evaluated in this article, or claim that may be made by its manufacturer, is not guaranteed or endorsed by the publisher.

## Footnotes

1
http://www.mbkbase.org/rice/

2
https://funricegenes.github.io/

3
https://www.ricedata.cn/gene/
