# Supplementary material for: CG and CHG Methylation Contribute to the Transcriptional Control of OsPRR37-Output Genes in Rice
Source: Front Plant Sci. 2022 Feb 15;13:839457. doi: 10.3389/fpls.2022.839457 (PMC8885545; doi:10.3389/fpls.2022.839457)
Supplement: Supplementary file 1 [file Data_Sheet_1.PDF]

SUPPLIMENTARY MATERIAL

Supplementary Figures

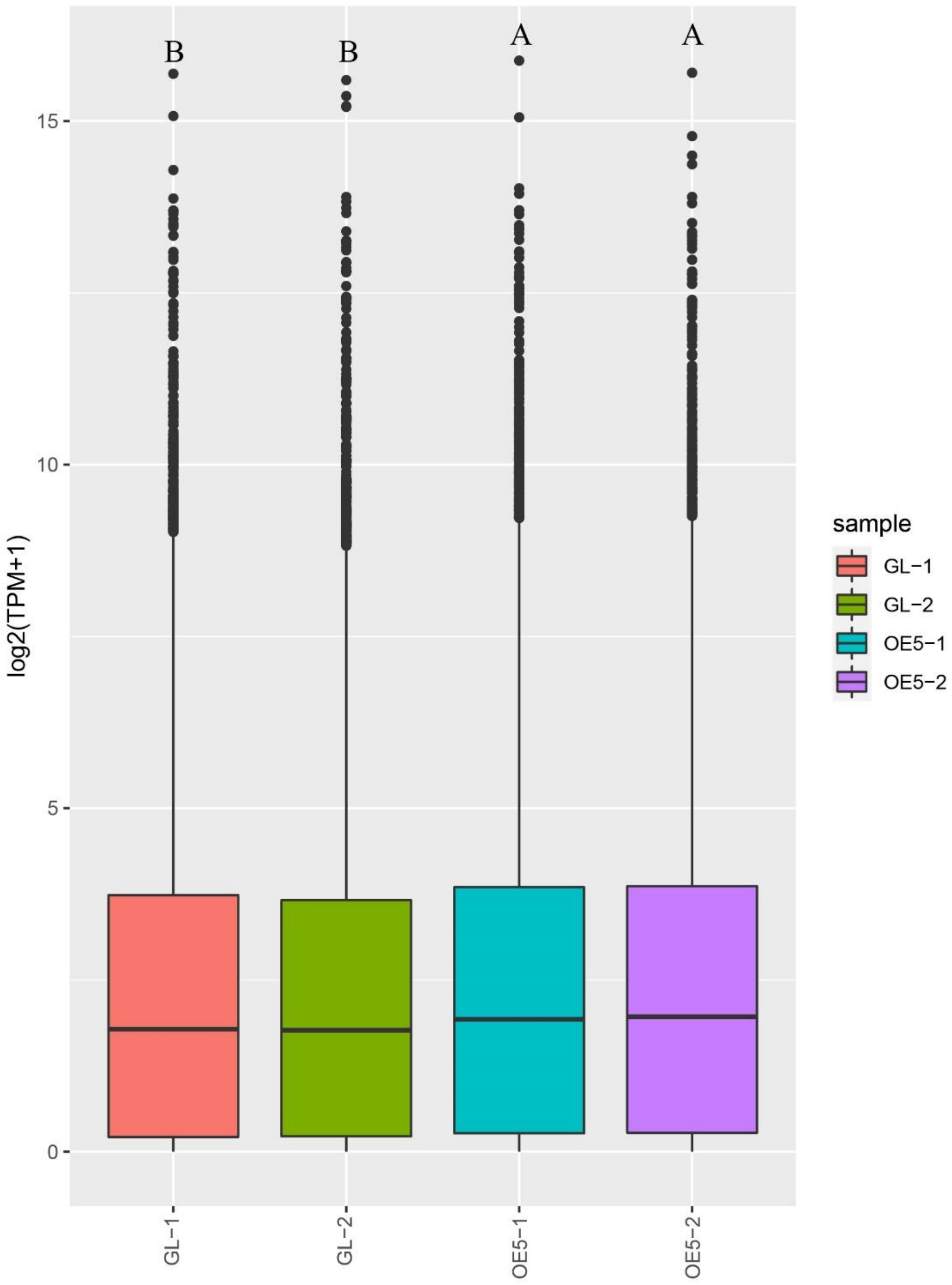

Supplementary Figure 1. Overall expression level of GL and OE5 samples. Different letters above violin plots represent significant differences at $P < 0.01$ as revealed by one-way ANOVA analysis (Tukey's multiple comparison test)

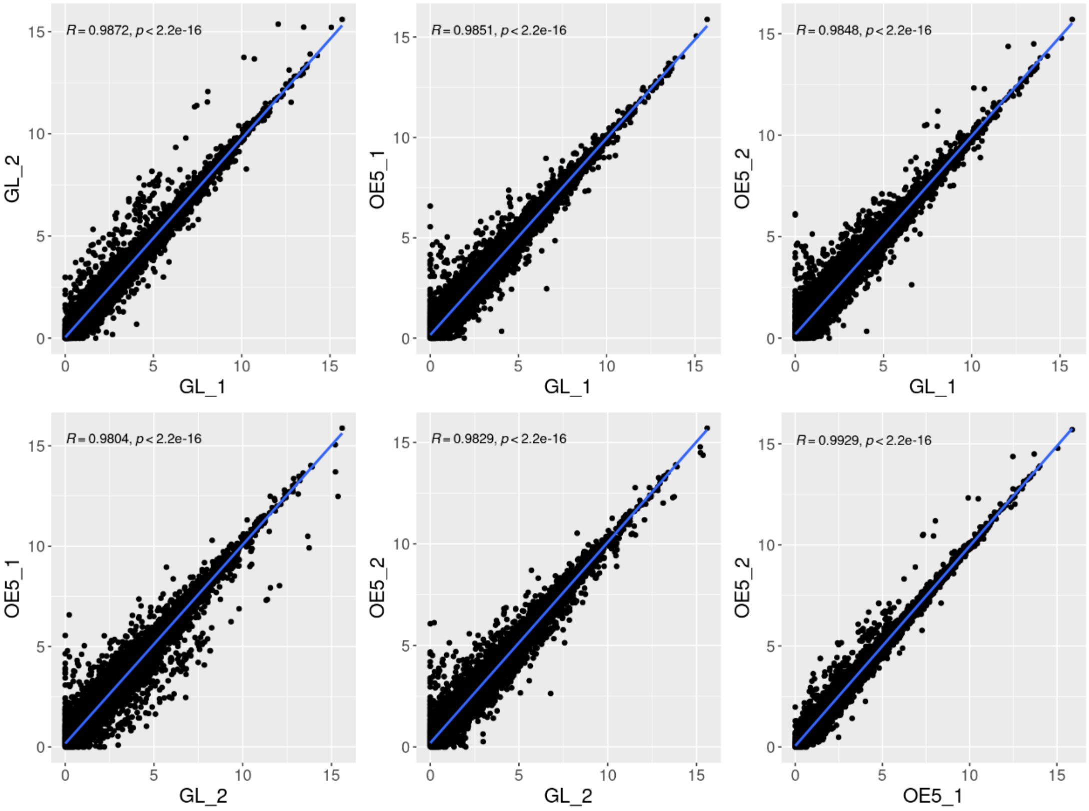

Supplementary Figure 2. Correlation of gene expression between RNA-seq samples

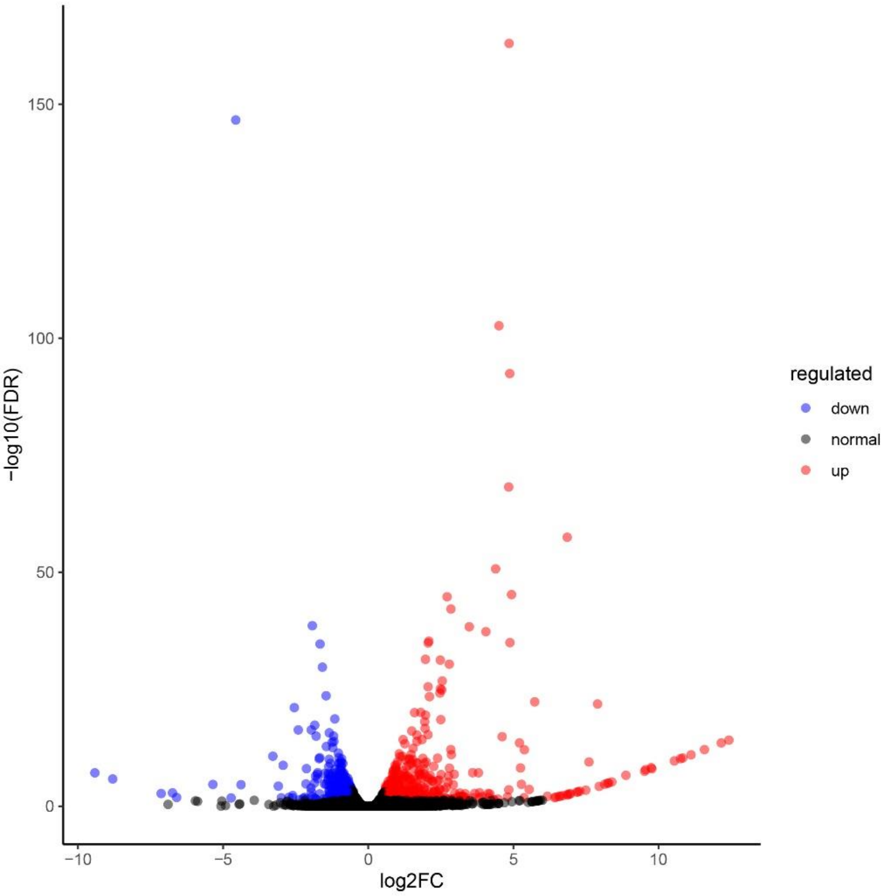

Supplementary Figure 3. Identification of differentially expressed genes between GL and OE5

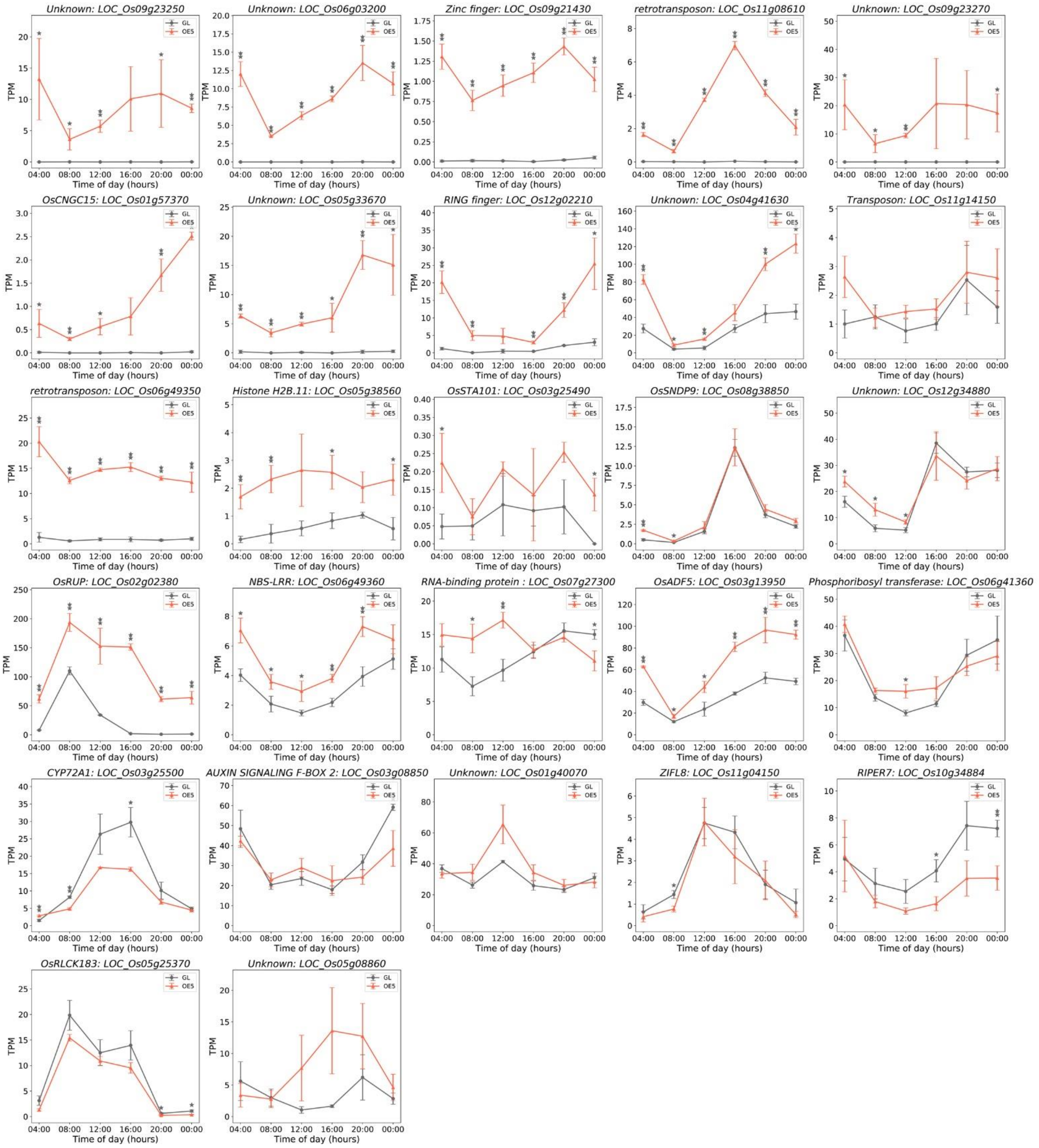

Supplementary Figure 4. Diurnal expression analysis of unknown overlapping genes. The asterisks above curves mean significance difference at $P < 0.05$ (one asterisk) and $P < 0.01$(two asterisks).

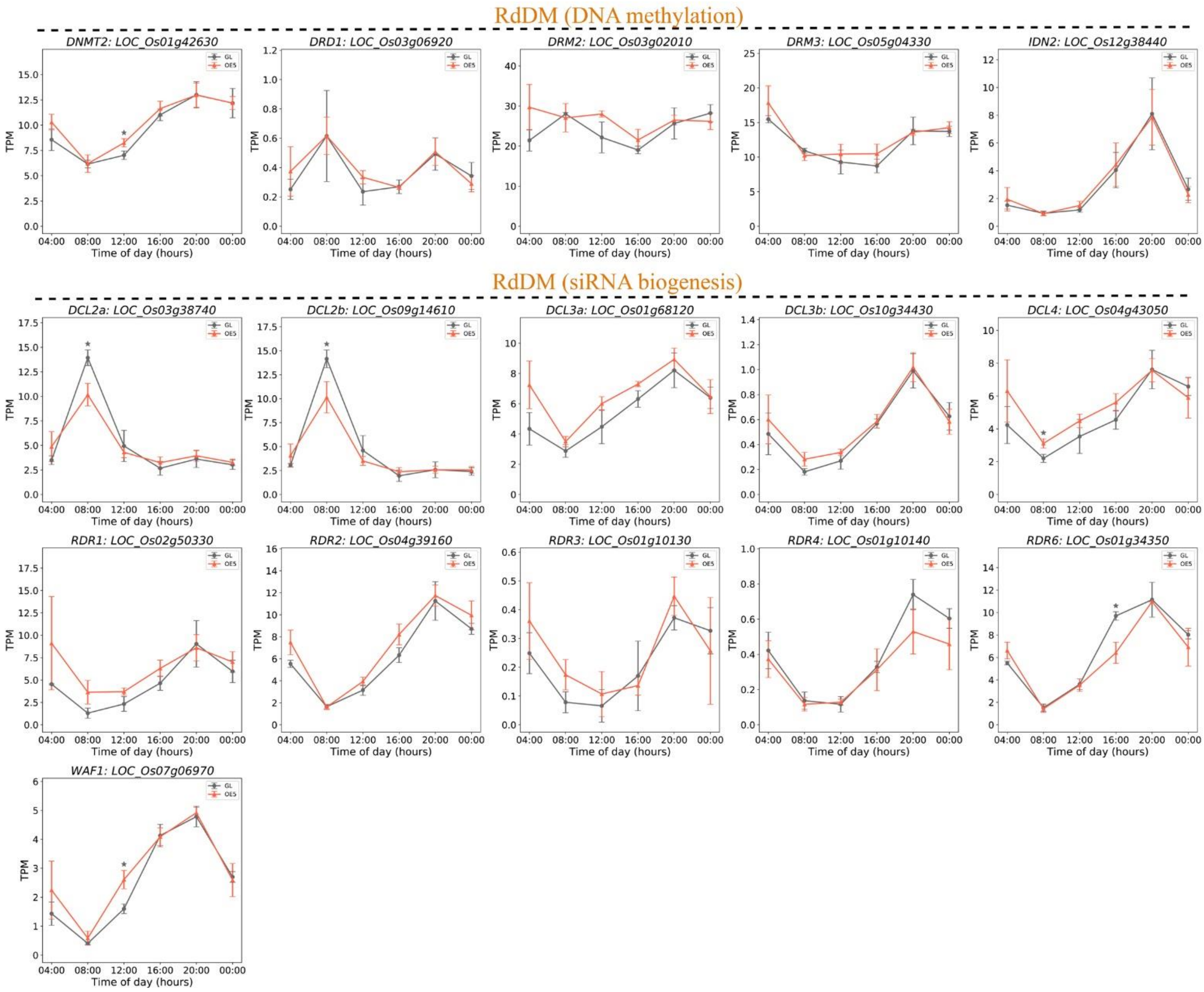

Supplementary Figure 5. Diurnal expression analysis of remained genes involved in RdDM pathway. The asterisks above curves mean significance difference at $P < 0.05$ (one asterisk) and $P < 0.01$(two asterisks).

Supplementary Tables

Supplementary Table 1. Tissue-specific sample information of microarray data used in this study

Sample_name	Developmental.stage	Tissue	Treatment	Planting.condition	Variety	Biological. replicates..variety	Technical.replicates. biological.replicate
germinating.seed	72 h after soaking in water	Germinating seed	NA	Laboratory	Zhenshan 97	2	NA
plumule.1	48 h after germination	Plumule	NA	Laboratory	Zhenshan 97	2	NA
plumule.2	48 h after germination	Plumule	Dark	Laboratory	Zhenshan 97	2	NA
radicle.1	48 h after germination	Radicle	NA	Laboratory	Zhenshan 97	2	NA
radicle.2	48 h after germination	Radicle	Dark	Laboratory	Zhenshan 97	2	NA
seedling.1	3 days after sowing	Seedling	NA	Hydroponics	Zhenshan 97	3	2
seedling.2	Trefoil stage	Seedling	NA	Hydroponics	Zhenshan 97	3	2
leaf.1	Young panicle (less than 1 mm)	Leaf	NA	Normal field	Zhenshan 97	2	NA
leaf.2	Young panicle: 40-50 mm	Leaf	NA	Normal field	Zhenshan 97	2	NA
leaf.3	5 days before heading	Flag leaf	NA	Normal field	Zhenshan 97	2	NA
leaf.4	14 days after flowering	Flag leaf	NA	Normal field	Zhenshan 97	2	NA
palea.lemma	1 day before flowering	Palea/lemma	NA	Normal field	Zhenshan 97	2	NA
root	Seedling with 2 tillers	Root	NA	Hydroponics	Zhenshan 97	2	NA
shoot	Seedling with 2 tillers	Shoot	NA	Hydroponics	Zhenshan 97	2	NA
sheath.1	Young panicle (less than 1 mm)	Sheath	NA	Normal field	Zhenshan 97	2	NA
sheath.2	Young panicle:40-50 mm	Sheath	NA	Normal field	Zhenshan 97	2	NA
stem.1	5 days before heading	Stem	NA	Normal field	Zhenshan 97	2	NA
stem.2	Heading stage	Stem	NA	Normal field	Zhenshan 97	2	NA
panicle.1	Young panicle (less than 1 mm)	Panicle	NA	Normal field	Zhenshan 97	3	2
panicle.2	Young panicle (3-5 mm)	Panicle	NA	Normal field	Zhenshan 97	3	2
panicle.3	Young panicle (10-15 mm)	Panicle	NA	Normal field	Zhenshan 97	3	2
panicle.4	Young panicle: 40-50 mm	Panicle	NA	Normal field	Zhenshan 97	2	NA
panicle.5	Heading stage	Panicle	NA	Normal field	Zhenshan 97	2	NA
spikelet	3 day after flowering	Spikelet	NA	Normal field	Zhenshan 97	2	NA
stamen	1 day before flowering	Stamen	NA	Normal field	Zhenshan 97	2	NA
endosperm.1	7 days after pollination	Endosperm(embryo is excluded)	NA	Normal field	Zhenshan 97	2	NA
endosperm.2	14 days after pollination	Endosperm(embryo is excluded)	NA	Normal field	Zhenshan 97	2	NA
endosperm.3	21 days after pollination	Endosperm(embryo is excluded)	NA	Normal field	Zhenshan 97	2	NA

Supplementary Table 2. Trimming statistics of Whole Genome Bisulfite Sequencing data

Sample	Raw reads	Raw bases(G)	Clean reads	Clean bases(G)	Clean rate(%)	Q30(%)	GC(%)	BS conversion rate(%)
GL-1	48,752,185	14.63	45,246,229	13.07	92.8	92.7	22.6	99.7
GL-2	63,773,173	19.13	59,675,582	17.4	93.6	92.6	23.3	99.6
OE5-1	59,530,161	17.86	56,065,476	16.4	94.2	92.8	23.0	99.8
OE5-2	56,527,166	16.96	53,133,046	15.52	94.0	92.6	22.8	99.7

Supplementary Table 3. Alignment of Whole Genome Bisulfite Sequencing data

Sample	Clean reads	Unique mapped reads	PCR duplications	Valid mapped reads
GL-1	45,246,229	24,162,833(53.4%)	5,068,357(21.0%)	19,094,476
GL-2	59,675,582	30,872,412(51.7%)	8,115,170(26.3%)	22,757,242
OE5-1	56,065,476	30,420,707(54.2%)	7,497,781(24.7%)	22,922,926
OE5-2	53,133,046	27,707,583(52.2%)	6,829,677(24.7%)	20,877,906

Supplementary Table 4. Sequencing depth and coverage of data

Sample	Context_type	Total_cytosine	Mean_depth	Effect_depth	Coverage	Coverage(depth>=5)
GL-1	CG	30,872,222	3.0	5.7	53.1%	14.0%
GL-1	CHG	27,422,379	3.1	5.3	58.5%	15.2%
GL-1	CHH	104,533,760	4.0	6.4	61.9%	19.0%
GL-2	CG	30,872,222	4.0	6.8	59.3%	17.4%
GL-2	CHG	27,422,379	4.1	6.3	64.6%	19.6%
GL-2	CHH	104,533,760	5.0	7.4	67.1%	23.2%
OE5-1	CG	30,872,222	4.0	6.9	57.1%	17.6%
OE5-1	CHG	27,422,379	4.1	6.5	62.6%	19.7%
OE5-1	CHH	104,533,760	5.1	7.7	65.5%	23.8%
OE5-2	CG	30,872,222	3.7	6.0	61.6%	17.9%
OE5-2	CHG	27,422,379	3.9	5.8	67.0%	21.3%
OE5-2	CHH	104,533,760	4.8	6.9	69.4%	25.4%

Supplementary Table 5. Global methylation levels for CG, CHG and CHH sites

Sample	mCG	mCG_percent	mCHG	mCHG_percent	mCHH	mCHH_percent
GL-1	3,272,054	10.6%	2,114,108	7.7%	4,185,459	4.0%
GL-2	3,942,658	12.8%	2,532,960	9.2%	4,681,130	4.5%
OE5-1	4,025,932	13.0%	2,649,978	9.7%	5,144,286	4.9%
OE5-2	3,793,694	12.3%	2,448,147	8.9%	4,684,537	4.5%

Supplementary Table 6. Quality control of RNA-seq clean data

Sample	Total read pairs	GC(%)	Clean base(bp)	Q20	Q30
GL-1	23,559,582	53	3,520,012,181	99.4%	85.6%
GL-2	25,090,638	54	3,748,219,768	99.4%	86.1%
OE5-1	25,898,111	53	3,871,921,883	99.3%	84.9%
OE5-2	26,350,196	53	3,938,070,846	99.4%	85.6%

Supplementary Table 7. Alignment of RNA-seq data

Samples	Total read pairs	Total mapped reads	Mapped ratio
GL-1	23,559,582	21,262,523	90.2%
GL-2	25,090,638	22,749,681	90.7%
OE5-1	25,898,111	23,316,069	90.0%
OE5-2	26,350,196	23,767,877	90.2%

Supplementary Table 8. Support data for Figure 5B

MSU_loci	Gene_symbol	Function	seqnames	width	methDiff	qval	CG_count	annotation	logFC
LOC_Os11g14150	Transposon	Putative	Chr11	228	-0.8111969	0.015738	9	Distal Intergenic	4.85326157
LOC_Os03g25490	OsSTA101	Putative	Chr3	467	-0.8565879	0.015738	6	Promoter (<=1kb)	2.08766517
LOC_Os02g02380	OsRUP	Putative	Chr2	107	0.7167148	0.025254	5	Promoter (1-2kb)	1.51398026
LOC_Os07g26540	OsHXK1	Known	Chr7	791	-0.8096712	0.015738	11	Promoter (<=1kb)	11.0994274
LOC_Os07g27300	RNA-binding protein	Putative	Chr7	288	-0.5977463	0.015738	12	Promoter (<=1kb)	0.95279263
LOC_Os04g41630	LOC_Os04g41630	Unknown	Chr4	128	-0.2387869	0.015738	17	Promoter (<=1kb)	5.21479448
LOC_Os09g23270	LOC_Os09g23270	Unknown	Chr9	94	-0.9304457	0.015738	7	Promoter (<=1kb)	9.82483102
LOC_Os01g57370	OsCNGC15	Putative	Chr1	90	-0.8534341	0.015738	8	Downstream (1-2kb)	9.56450513
LOC_Os12g34880	LOC_Os12g34880	Unknown	Chr12	132	-0.5613837	0.025254	9	Promoter (<=1kb)	1.56464942
LOC_Os05g33670	LOC_Os05g33670	Unknown	Chr5	221	-0.7177033	0.015738	10	Promoter (<=1kb)	8.27493171
LOC_Os06g44034	SDT/OsmiR156h	Known	Chr6	136	-0.4186401	0.048193	6	Promoter (2-3kb)	0.65142282
LOC_Os01g42160	MEGL14	Putative	Chr1	156	-0.6140306	0.015738	13	Promoter (2-3kb)	7.35808557
LOC_Os05g38560	Histone H2B.11	Putative	Chr5	147	-0.9481512	0.015738	7	Promoter (<=1kb)	2.99648095
LOC_Os03g08850	AUXIN SIGNALING F-BOX 2	Putative	Chr3	162	-0.7683079	0.025254	9	Promoter (<=1kb)	-0.69908188
LOC_Os06g41360	Phosphoribosyl transferase	Putative	Chr6	439	-0.4339326	0.015738	13	Promoter (<=1kb)	0.59901272
LOC_Os05g39540	OsZIP9	Known	Chr5	783	-0.6548336	0.015738	10	Promoter (<=1kb)	2.85065752

Supplementary Table 9. Support data for Figure 5C

MSU_loci	Gene_symbol	Function	seqnames	width	methDiff	qval	CHG_count	annotation	logFC
LOC_Os06g49350	retrotransposon	Putative	Chr6	260	-0.4446391	0.0363154	10	Promoter (<=1kb)	4.5408213
LOC_Os11g14150	Transposon	Putative	Chr11	1357	-0.2050417	0.0041513	37	Promoter (<=1kb)	4.8532616
LOC_Os03g25490	OsSTA101	Putative	Chr3	94	-0.4999562	0.0108765	9	Distal Intergenic	2.0876652
LOC_Os05g25370	OsRLCK183	Putative	Chr5	333	0.20907248	0.0067947	9	5' UTR	-1.790562
LOC_Os12g02210	RING finger	Putative	Chr12	234	-0.4298426	0.0052555	6	Promoter (1-2kb)	5.2435229
LOC_Os09g23250	LOC_Os09g23250	Unknown	Chr9	174	-0.2246218	0.0247961	6	Promoter (1-2kb)	11.618061
LOC_Os07g26540	OsHXK1	Known	Chr7	1084	-0.24092	0.0085022	13	Promoter (<=1kb)	11.099427
LOC_Os06g03200	LOC_Os06g03200	Unknown	Chr6	136	-0.3014988	0.0114301	5	Promoter (<=1kb)	10.865097
LOC_Os09g21430	Zinc finger	Putative	Chr9	536	0.23874443	0.0041513	12	Intron	10.779811
LOC_Os11g08610	retrotransposon	Putative	Chr11	786	-0.4212087	0.0041513	20	Exon	10.586134
LOC_Os11g04150	ZIFL8	Putative	Chr11	97	-0.3147427	0.0164331	6	Promoter (<=1kb)	-1.010984
LOC_Os01g40070	LOC_Os01g40070	Unknown	Chr1	104	-0.3615598	0.0085022	7	Distal Intergenic	-0.978161
LOC_Os08g38850	OsSNDP9	Putative	Chr8	485	-0.4894586	0.0041513	10	Promoter (<=1kb)	1.6509955
LOC_Os05g33670	LOC_Os05g33670	Unknown	Chr5	207	-0.4895094	0.0224495	5	Promoter (<=1kb)	8.2749317
LOC_Os05g08860	LOC_Os05g08860	Unknown	Chr5	588	0.24844012	0.0199979	15	Promoter (2-3kb)	-2.173924
LOC_Os10g34884	RIPER7	Putative	Chr10	4936	0.22741254	0.0041513	146	Promoter (1-2kb)	-1.226017
LOC_Os03g13950	OsADF5	Putative	Chr3	850	-0.4183993	0.0041513	10	Downstream (2-3kb)	0.6716088
LOC_Os07g48980	OsNAS3	Known	Chr7	618	-0.3611991	0.048438	6	Distal Intergenic	-1.961784
LOC_Os07g41370	OsMADS18	Known	Chr7	158	-0.2512089	0.0393703	10	Distal Intergenic	-0.650052
LOC_Os05g38560	Histone H2B.11	Putative	Chr5	211	-0.4749849	0.0049426	8	Promoter (<=1kb)	2.9964809
LOC_Os03g25500	CYP72A1	Putative	Chr3	244	-0.3793179	0.0067947	8	Promoter (2-3kb)	-0.659127
LOC_Os01g46860	PHT1-11	Known	Chr1	126	0.45126216	0.0174635	6	Promoter (1-2kb)	1.1691837
LOC_Os03g24930	OsRLCK109	Known	Chr3	70	-0.2567508	0.0088317	6	Distal Intergenic	-0.600082
